# Retinal prolactin isoform *PRLΔE1* sustains rod disease in inherited retinal degenerations

**DOI:** 10.1038/s41419-024-07070-1

**Published:** 2024-09-18

**Authors:** Raghavi Sudharsan, Jennifer Kwok, Malgorzata Swider, Alexander Sumaroka, Gustavo D. Aguirre, Artur V. Cideciyan, William A. Beltran

**Affiliations:** 1 Division of Experimental Retinal Therapies, Department of Clinical Sciences & Advanced Medicine, School of Veterinary Medicine, University of Pennsylvania, Philadelphia, PA USA; 2 Center for Hereditary Retinal Degenerations, Scheie Eye Institute, Department of Ophthalmology, University of Pennsylvania Perelman School of Medicine, Philadelphia, PA USA

**Keywords:** Retina, Neurodegeneration

## Abstract

*PRLΔE1*, a retina-specific isoform of prolactin, is expressed in multiple and diverse forms of canine inherited retinal degeneration (IRD). We find that while *PRLΔE1* expression in rods is not associated with the initial phase of disease characterized by acute photoreceptor cell death, it is associated with the protracted phase of slow cell loss. Restoration of photoreceptors to a healthy state by gene-specific replacement therapy of individual IRDs successfully suppresses *PRLΔE1* expression. Moreover, short-term *PRLΔE1* silencing using shRNA results in preservation of outer nuclear layer thickness, suggesting *PRLΔE1* drives retinal disease. However, longer-term observations reveal off-target toxic effects of the *PRLΔE1* shRNA, precluding determination of its full therapeutic potential. Future research efforts aimed at enhancing the safety and specificity of *PRLΔE1*-targeting strategies may identify a potential universal intervention strategy for sustaining photoreceptors during the prolonged phase of multiple IRDs.

## Introduction

Prolactin (PRL) is a pleiotropic hormone that contributes to multiple physiologic and homeostatic functions in the body including maturation of mammary glands and initiation of lactation, metabolic regulation, angiogenesis, immunomodulation, neuroprotection, and neurogenesis [1–6]. Full-length PRL activates the JAK/STAT pathway through its transmembrane receptor PRL-R, which is expressed on multiple cell types [7]. PRL plays a protective role in light-induced retinal degeneration and in diabetic retinopathy [3, 8–12]. Its anti-apoptotic and antioxidant effects promote survival of both the photoreceptors (PR) and the retinal pigment epithelium (RPE) [10, 13, 14]. PRL modulates neurotrophin signaling from glial cells to protect PRs from light-induced damage in the rodent retina [10]. Clinically, elevated circulating levels of PRL have been associated with a reduced risk of proliferative diabetic retinopathy in diabetic patients, largely due to the antiangiogenic properties of vasoinhibins, a family of bioactive peptides generated from the N-terminus of PRL by proteolytic cleavage. These peptides are currently being investigated as a therapeutic target to regulate ocular angiogenesis and prevent disease progression in diabetic retinopathy and diabetic macular edema [9, 15–18].

Although mainly synthesized by lactotrophic cells of the anterior pituitary gland, PRL is also expressed in other tissues [19, 20], including normal retinas from rats, green monkeys, and baboons [15, 21, 22]. Notably, we previously identified *PRLΔE1*, a novel isoform of *PRL* that lacks the first exon, expressed in the retinas of two non-allelic, naturally occurring canine models of early-onset inherited retinal degeneration (IRD), *PDE6β*-RCD1 and *RPGR*-XLPRA2 [23]. In these two models, a large number of PRs die acutely between 5 and 7 weeks of age, marking the early phase of disease [24, 25]. Immediately after this burst of cell death, *PRLΔE1* expression is induced in the remaining rods and increases through the protracted phase of disease/degeneration, even as the rate of cell death declines [23]. Expression of *PRLΔE1* is not observed in healthy young or adult dog retinas. However, this *PRL* isoform is found in the aged human retina [23].

In the current study, we have further examined the expression of *PRLΔE1* in two additional canine models of IRD. We demonstrate that *PRLΔE1* expression correlates with protracted RD and not acute PR cell death. We also provide preliminary insights into the function of *PRLΔE1* in IRD and present evidence that *PRLΔE1* is a viable therapeutic target for promoting PR cell survival.

## Results

### Rod-specific expression of *PRLΔE1* in canine IRD with protracted PR degeneration

**Fig. 1:**
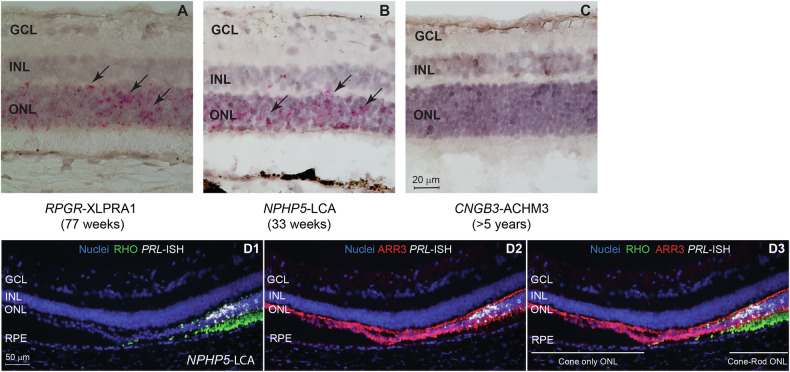
*PRLΔE1* expression in two additional canine models of inherited retinopathies and specifically in rods. *PRLΔE1* mRNA identified by RNA in situ hybridization (magenta punctate staining, see black arrows) in the PRs in *RPGR*-XLPRA1 (**A**) and *NPHP5*-LCA (**B**), but not in *CNGB3*-ACHM3, a cone-specific disease (**C**). **D1**–**D3** *PRLΔE1* expression observed by RNA-ISH (white) only in the degenerating *NPHP5*-LCA ONL containing both rods and cones (**D1**, **D3**) and not in the adjacent cone-only ONL (**D2**, **D3**). Rhodopsin (RHO) staining for rods in green, Arrestin-3 (ARR3) staining for cones in red. Each panel represents observations from a single dog for the specified genotype. ONL outer nuclear layer, INL inner nuclear layer, GCL ganglion cell layer.

In our earlier publication [23], we described the expression of *PRLΔE1* transcript in the PRs in *PDE6β*-RCD1 [26–28] and *RPGR*-XLPRA2 [24, 29] dogs. To determine whether *PRLΔE1* expression is common to other forms of IRD, we examined additional canine models of IRD. We now show that this shorter *PRL* transcript is also expressed in *RPGR*-XLPRA1 (X-Linked Progressive Retinal Atrophy 1, caused by a five-nucleotide deletion in the *RPGR* exon ORF15) [29, 30] (Fig. 1A) and *NPHP5*-LCA (Leber Congenital Amaurosis, caused by a single nucleotide insertion in exon 10 of the *IQCB1*/*NPHP5* gene) [31, 32] (Fig. 1B). Both diseases affect rods and cones, however, with varying age of onset and disease severity. In the rapidly degenerating *NPHP5*-LCA model, *PRLΔE1* transcript was detected at 14 weeks of age. This time point is characterized by early stages of PR loss and moderate thinning of the ONL, consistent with the early phase of degeneration in this model [31]. In contrast, in the *RPGR*-XLPRA1 model, degenerative changes in the PR are observed before onset of cell death [33]. *PRLΔE1* expression was observed starting at 26 weeks of age, which notably precedes the onset of noticeable PR loss. This early upregulation of *PRLΔE1* expression prior to extensive PR cell death, indicates a potential role in the initial response to degenerative stress (Supplementary Fig. 1).

To determine whether PR dysfunction without ongoing degeneration also induces *PRLΔE1* expression, we examined a model of achromatopsia (*CNGB3*-ACHM3; mutation in Cyclic Nucleotide Gated Channel Subunit Beta 3), a disease with impaired cone function and structure, but no rod degeneration [34, 35]. Notably, in the absence of rod degeneration, retinal *PRLΔE1* expression was not observed (Fig. 1C). Taken together, these results suggest that *PRLΔE1* expression is associated with ongoing rod disease.

Identification of PR cell subclass (rods and/or cones) that express *PRLΔE1* by RNA-in situ hybridization (RNA-ISH) within the outer nuclear layer (ONL, the cellular layer containing the PR cell bodies) is challenging due to the dense packing of rod and cone nuclei in this area. To determine the PR cell type that specifically expresses *PRLΔE1*, we took advantage of *NPHP5*-LCA retinas in which degeneration results in adjacent areas containing both rods and cones, or only cones. *PRLΔE1* RNA-ISH staining was found in regions containing both PR cell types but not in cone-only areas (Fig. 1D), supporting rod-specific expression in degenerating retina. We further confirmed *PRLΔE1* expression in rods, and not cones, using data from single-cell RNAseq analysis. (Supplementary Fig. 2). Thus, our study now firmly establishes the expression of *PRLΔE1* in diseased rods in four different canine models of IRD.

### *PRLΔE1* expression colocalizes with areas of PR disease in *RPGR*-XLPRA carriers

**Fig. 2:**
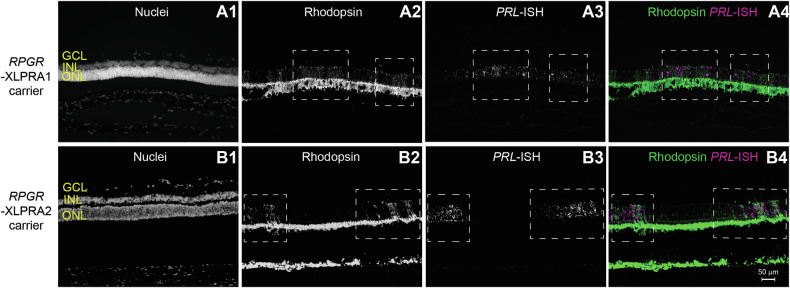
*PRLΔE1* expression overlaps with areas of PR degeneration in carriers of *RPGR* mutations. **A1**, **B1** Retinal cellular layers identified by Hoechst staining of nuclei. Patchy areas of degeneration (white dashed boxes) resulting from random X-inactivation identified by rhodopsin mislocalization to PR cell bodies in **A2** *RPGR*-XLPRA1 carriers (age: 24 weeks) and **B2** *RPGR*-XLPRA2 (age: 24 weeks) carrier female dogs. **A3**, **B3** *PRLΔE1* transcript identified by RNA-ISH. **A4**, **B4** Merged image show rhodopsin immunostaining in green and *PRLΔE1* in magenta. Retinas from one *RPGR*-XPRA1 carrier dog and two *RPGR*-XLPRA2 carrier dogs were examined. ONL outer nuclear layer, INL inner nuclear layer, GCL ganglion cell layer.

To determine whether *PRLΔE1* expression is restricted to degenerating rods, we examined its expression in the two *RPGR*-XLPRA carriers, XLPRA1 and XLPRA2. In female dogs with a single mutant copy of *RPGR*, random X-chromosome inactivation creates a mosaic of normal and mutant PRs [36]. Consequently, degeneration within the ONL occurs in patches that can be identified by opsin mislocalization to the PR cell bodies [36]. In both carrier retinas, *PRLΔE1* expression overlapped with the patchy areas of degeneration identified by rhodopsin mislocalization and was not observed in the intervening patches with normal PRs (Fig. 2). Thus, *PRLΔE1* expression is limited to mutant rods with active disease.

### *PRLΔE1* expression is not directly associated with acute PR cell death

**Fig. 3:**
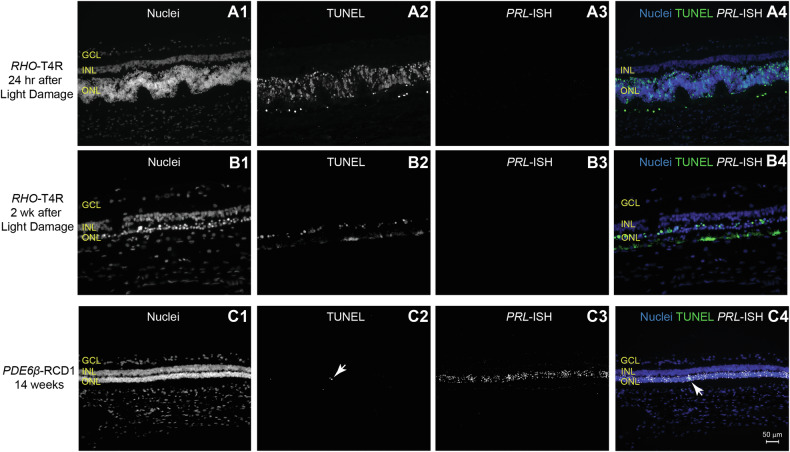
*PRLΔE1* expression is not associated with acute PR cell death in *RHO*-T4R ADRP but correlates with protracted rod degeneration. *PRLΔE1* expression is absent in acute light-induced RD in *RHO*-T4R retinas 24 h and 2 weeks after light-induced damage but is observed in the more slowly degenerating PDE*6β*-RCD1 retina (age: 14 weeks). **A1**, **B1**, **C1** Retinal nuclear layers identified by Hoechst staining. **A2**, **B2**, **C2** TUNEL staining. **A3**, **B3**, **C3** *PRL*-ISH. **A4**, **B4**, **C4** Merged images show nuclei in blue, TUNEL staining in green, and *PRLΔE1* in white. Retinas from one *RHO*-T4R dog for each time point and one *PDE6β*-RCD1 dog were examined. ONL outer nuclear layer, INL inner nuclear layer, GCL ganglion cell layer.

To assess whether *PRLΔE1* is associated with PR cell death, we examined its expression in the *RHO-T4R* dog, a large animal model of acute light-induced rod cell death caused by the T4R mutation in rhodopsin [37, 38]. In this model, rapid rod OS fragmentation occurs within hours of a one-minute exposure to white light at a corneal irradiance of 1 mW/cm^2^, and significant rod cell death is observed by extensive TUNEL labeling in the ONL at 24 h [39, 40]. However, no *PRLΔE1* expression was detected at this time point (Fig. 3A). By 2 weeks post light exposure, the retina was reduced to one to two rows of PR with ongoing cell death, yet no *PRLΔE1* was observed (Fig. 3B). In contrast, the *PDE6β*-RCD1 model, characterized by a protracted disease course, showed extensive *PRLΔE1* expression at 14 weeks of age, a time when minimal TUNEL staining was present (Fig. 3C).

To further explore the dynamics of *PRLΔE1* expression in the *RHO*-T4R light-sensitive dog, we assessed its levels following exposure to two different light exposure intensities (0.5 mW/cm^2^ and 0.3 mW/cm^2^ for 1 min). These lower light intensities result in fewer TUNEL-labeled cells at 24 h post exposure and a greater survival of PRs at the two-week mark, though cell death is ongoing [39]. Nonetheless, *PRLΔE1* was not expressed in the PRs at any time point under these conditions (Supplementary Fig. 3). Thus, our results suggest that *PRLΔE1* expression is not associated with the acute rod cell death that can be experimentally triggered in this model but is instead linked to the chronic degenerative state observed in models with prolonged disease.

### *PRLΔE1* expression is suppressed in *RPGR* and *NPHP5* mutant retinas after corrective gene augmentation therapy

**Fig. 4:**
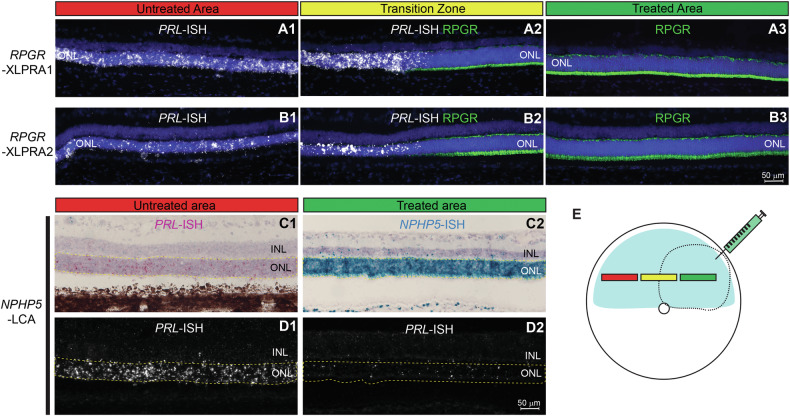
Specific corrective gene augmentation therapy is associated with suppression of *PRLΔE1* expression in treated areas. **A1**–**A3** *PRLΔE1* RNA-ISH in a *RPGR*-XLPRA1 dog (treated at 28 weeks of age, assessed 49 weeks post-treatment) shows *PRLΔE1* expression in the untreated retina (**A1**) but the transcript is not expressed in AAV-*RPGR* treated area (**A3**). **B1**–**B3** *PRLΔE1* RNA-ISH in a *RPGR*-XLPRA2 (treated at 5 weeks of age, assessed 33 weeks post-treatment) dogs shows *PRLΔE1* expression in the untreated retina (**B1**) but none in the AAV-RPGR treated area (**B3**). **A2**, **B2** Transition zone presents a clear demarcation in *PRLΔE1* expression between treated and untreated regions. *PRLΔE1* RNA-ISH: White punctate staining; RPGR immunostaining in green. **C1**–**C2**, **D1**–**D2** *PRLΔE1* RNA-ISH in a NPHP5-LCA dog (treated at 6 weeks of age, assessed 27 weeks post-treatment) shows abundant *PRLΔE1* in the ONL of the untreated area (**C1**, **D1**) but markedly reduced levels in the AAV-*NPHP5* treated region (**C2**, **D2**). *PRLΔE1* and *NPHP5* RNA-ISH in pink (Fast Red) and green (HRP-Green) staining, respectively in (**C1**) and (**C2**) observed by brightfield microscopy. Only Fast Red dye, and thus *PRLΔE1* RNA-ISH, staining is observed by fluorescence microscopy (**D1**, **D2**) in the same regions as panel (**C**). **E** Schematic representation of the *en face* fundus image of the retina, dotted line corresponds to the area of treatment (bleb) injected with a corrective gene therapy AAV vector. A single retina for each specified genotype was examined. ONL outer nuclear layer, INL inner nuclear layer.

Since we found that *PRLΔE1* expression was restricted to unhealthy/diseased rods, we predicted that *PRLΔE1* expression would be downregulated or abrogated if degeneration was halted, and PR homeostasis restored therapeutically. To test this hypothesis, we examined *PRLΔE1* expression in two non-allelic diseases (*RPGR*-XLPRA and *NPHP5*-LCA) that have been previously successfully treated by AAV-mediated gene augmentation therapy [33, 41, 42]. Notably, in stark contrast to the high level of expression in untreated regions in retinas of the two *RPGR*-XLPRA models (XLPRA1 and XLPRA2), *PRLΔE1* was not expressed within the AAV-*RPGR* treated area (treated at 28 weeks and 5 weeks of age, assessed at 49 and 33 weeks post treatment, respectively) (Fig. 4A, B). Similarly, in the *NPHP5*-LCA retinas, *PRLΔE1* expression was remarkably reduced within the AAV-*NPHP5* treated (at 6 weeks of age) retinal area when assessed 27 weeks post treatment, compared to the untreated retina (Fig. 4C, D). As expression of *PRLΔE1* is associated with rod disease in the protracted phase of IRDs and can be suppressed by restoring PR to normal state following gene therapy, it raised the intriguing possibility that *PRLΔE1* itself could be contributing to disease.

### Knockdown of *PRLΔE1* expression confers transient protection to photoreceptors

To determine if *PRLΔE1* expression is a cause or an effect of the disease process, we used RNA interference to knock down *PRLΔE1* expression in *PDE6β*-RCD1 and *RPGR*-XLPRA2 retinas. Three different *PRLΔE1*-targeting shRNAs driven by the ubiquitous H1 promoter were tested in vitro in HEK293 cells co-transfected with a plasmid expressing *PRLΔE1* (Supplementary Fig. 4). The shRNA showing the highest efficacy (shRNA2, ~70% knockdown) was selected for packaging in AAV2/5 vector for sustained expression after subretinal delivery in canine retinas. AAV2/5-shRNA_*PRLΔE1*_ was delivered subretinally in one *PDE6β*-RCD1 (age: 5 weeks) and two *RPGR*-XLPRA2 (age: 7 weeks) dogs at ages corresponding to the early acute phase of cell death and start of *PRLΔE1* expression in surviving PRs in these models [23–25].

**Fig. 5:**
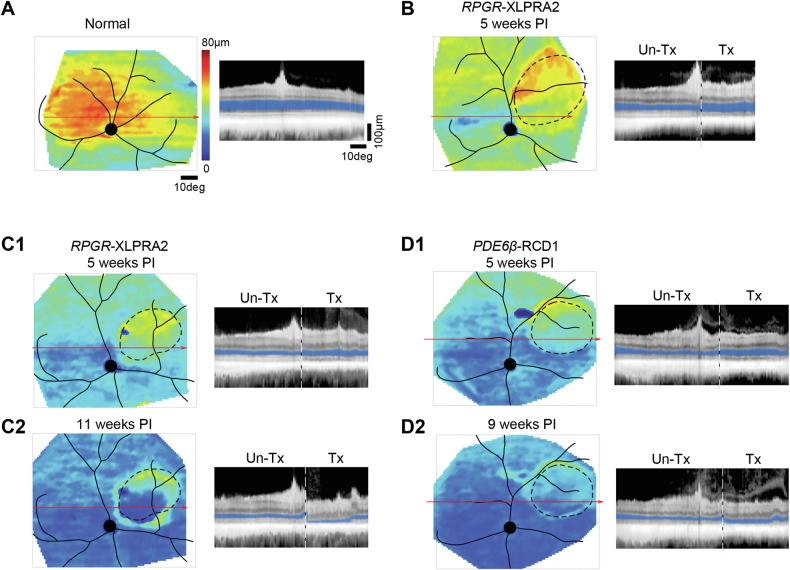
In vivo assessment of ONL thickness after treatment with shRNA_*PRLΔE1*_ in *PDE6β*-RCD1 and *RPGR*-XLPRA2 retinas. **A**–**D2** Topographical pseudocolor maps (left) and reconstituted horizontal b-scans spanning through (red arrow) the treated (Tx) and untreated (Un-Tx) retinal areas (right) of a normal untreated retina (**A**), two *RPGR*-XLPRA2 treated retinas (**B**, **C1**–**C2**), and a *PDE6β*-RCD1 treated retina (**D1**–**D2**). Dashed lines on maps (left) encircle the area of the subretinal bleb, dashed vertical lines on b-scans (right) demarcate the transition between Tx and Un-Tx. ONL layer thickness on b-scans is highlighted in blue. Calibration bars and pseudocolor scale shown in (**A**) are applicable to all panels.

To assess the impact of shRNA-mediated *PRLΔE1* knockdown on retinal structure and gene expression, we used in vivo imaging and histochemical analysis at two time points. Confocal scanning laser ophthalmoscopy and spectral domain optical coherence tomography (cSLO/OCT) were utilized to monitor retinal structure. In normal retinas, a hyporeflective layer corresponding to the ONL was evident in cross-sectional cSLO/OCT images (Fig. 5A). Quantitative ONL thickness maps were generated across wide expanses of the retina. These maps revealed preserved ONL in the treated areas for all three dogs at 5 weeks post injection (PI) (Fig. 5B, C1, D1). The ONL was thicker in the treated regions, clearly demarcated from untreated areas both on topographic representation as well as on representative cross-sections (Fig. 5B, C1, D1). However, we noted a thinning of the inner segment/outer segment (IS/OS or EZ) layer within the treated areas. One *RPGR*-XLPRA2 dog was terminated at this point for structural/expression analysis. For extended evaluation, cSLO/OCT imaging was continued up to 9 weeks PI in the *PDE6β*-RCD1 dog and up to 11 weeks PI in the remaining *RPGR*-XLPRA2 dog. Over time, there appeared to be progressive thinning of the ONL layer both in treated and untreated areas (Fig. 5C2, D2), prompting termination of the study at these time points.

**Fig. 6:**
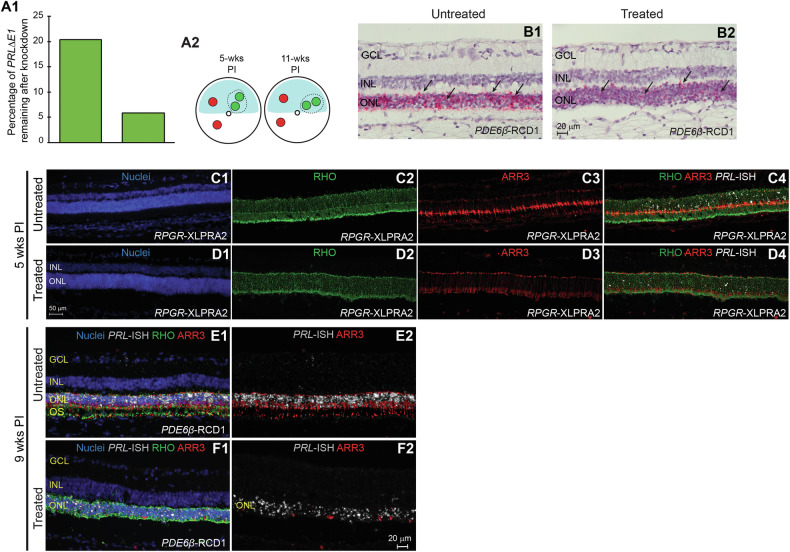
Assessment of AAV2/5- shRNA_*PRLΔE1*_-mediated *PRLΔE1* knockdown and effect on photoreceptors. **A1** Percentage of *PRLΔE1* remaining as quantified by qPCR in *RPGR*-XLPRA2 retinas 5 weeks and 11 weeks post injection (PI). **A2** Schematic representation of *RPGR*-XLPRA2 retinas with treated area demarcated by dotted lines and 3 mm retinal punches used for qPCR analysis shown with green (treated area) or red (untreated area) circles. Representative RNA-ISH (magenta dots, arrows) image showing *PRLΔE1* expression in ONL of untreated (**B1**) and shRNA_*PRLΔE1*_-treated (**B2**) retinal areas in *PDE6β*-RCD1. **C1**–**D4** Immunohistochemical staining for rods (Rhodopsin, RHO, green) and cones (Arrestin-3, ARR3, red) with *PRLΔE1*-ISH (white) in untreated (**C1**–**C4**) and treated (**D1**–**D4**) regions of a *RPGR*-XLPRA2 retina 5-weeks PI. **E1**–**F2** Immunohistochemical staining for rods (Rhodopsin, RHO, green) and cones (Arrestin-3, ARR3, red) with *PRLΔE1*-ISH (white) in untreated (**E1**–**E2**) and, treated (**F1**–**F2**) regions of a *PDE6β*-RCD1 retina 9 weeks PI. Note: Only *PRLΔE1* and ARR3 are shown in (**E2**) and (**F2**) to emphasize the loss of cones in the retina following the shRNA treatment. Data presented is from one *PDE6β*-RCD1 and 2 *RPGR*-XLPRA2 dogs treated with shRNA_*PRLΔE1*_. ONL outer nuclear layer, INL, inner nuclear layer, GCL ganglion cell layer.

**Fig. 7:**
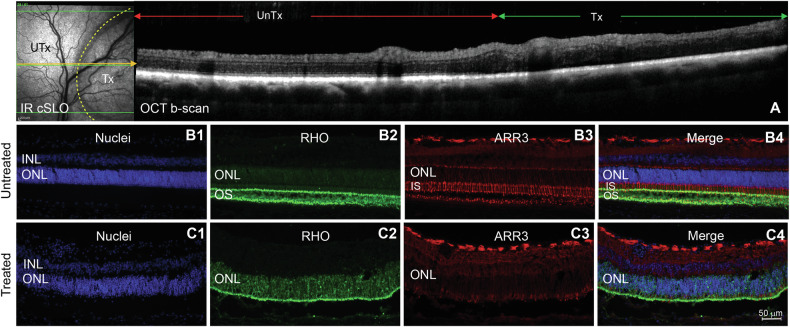
Effect of subretinal injection of AAV2/5-shRNA_*PRLΔE1*_ in normal dog retina. **A** cSLO *en face* image and OCT b-scan of the retina showing the treated (Tx) and the untreated (UnTx) areas. **B1**–**C4** Immunohistochemical staining of rods (RHO, green) and cones (ARR3, red) in untreated (**B1**–**B4**) and treated (**C1**–**C4**) retinal areas 8 weeks post injection. Mislocalization of Rhodopsin (RHO) protein to the cell bodies (**C2**) and loss of cone specific Arrestin-3 (ARR3) staining (**C3**) is observed in the treated retina. OS outer segment layer, IS inner segment layer, ONL outer nuclear layer, INL inner nuclear layer.

Quantitative-PCR analysis showed approximately 80% knockdown at 5-weeks PI and nearly 95% reduction in *PRLΔE1* mRNA at 11-weeks PI in the shRNA treated area in the two *RPGR-*XLPRA2 dogs (Fig. 6A). This reduction was confirmed by RNA-ISH in the *PDE6β*-RCD1 dog (Fig. 6B). Immunohistochemistry performed in the *RPGR*-XLPRA2 dog terminated at 5 weeks PI confirmed that the ONL was thicker in the shRNA-treated area compared to the untreated areas. *PRL*-ISH indicated a qualitative reduction in *PRLΔE1* expression in the treated area where the ONL thickness was maintained. However, knockdown of *PRLΔE1* was associated with a shortening of IS and OS and did not correct opsin mislocalization in rods. Surprisingly, in the dogs treated for longer duration, we noted a loss of arrestin-3 (ARR3) staining, a marker of cone PRs, within the treated area, suggesting that *PRLΔE1* knockdown caused a loss of cone cell viability (Fig. 7C, D). Thus, while shRNA_*PRLΔE1*_ mediated *PRLΔE1* knockdown was protective at first, its longer-term protection was compromised by an unexpected loss of cones in treated retinas.

### *PRLΔE1* shRNA has a potential non-specific toxic effect that is detrimental to photoreceptor survival

Since cones do not express *PRLΔE1*, we hypothesized that cone cell death following shRNA treatment was due to either the loss of *PRLΔE1* in the diseased rods, or a non-specific effect of this particular shRNA_*PRLΔE1*_. To distinguish between these two possibilities, we subretinally injected a normal adult dog with AAV2/5-shRNA_*PRLΔE1*_ at the same dose used in the mutant dogs. We presumed that since rods in the normal retina do not express *PRLΔE1*, shRNA injection would have no effect. However, at 8 weeks PI, we observed a disruption in the ONL and loss of the IS/OS line by in vivo OCT imaging, as seen in the mutants (Fig. 7A). After termination, we also observed mislocalization of rod opsin and loss of cones (Fig. 7B, C). These results suggest that the cone cell death observed in the normal and mutant retinas after shRNA treatment is not due to silencing of *PRLΔE1* in rods but is instead due to a non-specific toxic effect of our current shRNA. As *PRLΔE1* knockdown early after injection preserved the ONL, future studies are warranted to determine if therapeutic targeting of *PRLΔE1* has the potential to be protective in multiple forms of IRD when implemented with an improved gene silencing strategy.

## Discussion

A defining feature of most IRDs is that despite being genetically identical, PRs do not die at the same time. Instead, as is observed in many animal models of RD [24, 25, 43, 44], there is a surge in PR cell death in the early stages of disease followed by a protracted phase of PR cell loss that provides a potential window for therapeutic intervention. Notably, this initial burst of cell death also promotes transcriptional changes in the surviving PRs that leads to upregulation of both pro-survival and pro-inflammatory molecules [24, 45–47], the balance of which may determine the fate of each PR in the later phase of degeneration. To identify factors that impact PR survival during the protracted phase of disease, we analyzed retinal transcripts and pathways that are altered in two early onset canine diseases, *PDE6β*-RCD1 and *RPGR*-XLPRA2, when more than 50% of the PRs are lost [46]. One of the top upregulated transcripts in both diseases was *PRL* [46] which we subsequently showed to be the novel *PRLΔE1* isoform [23]. Furthermore, our previous studies showed that neither the full-length PRL nor the short isoform is expressed in normal retinas (Sudharsan et al. [23], Supplementary Fig. 1) [23].

In the current study*, PRLΔE1* was consistently upregulated in rods during chronic stages of disease progression across several IRDs. This expression pattern implies that *PRLΔE1* may play a role in the cellular response to prolonged stress and degeneration rather than being a direct mediator of cell death. The early preservation of ONL following *PRLΔE1* knockdown further supports the notion that *PRLΔE1* is not merely a byproduct of degeneration but may actively contribute to the progression of the disease. Its expression during the chronic phase of degeneration hints at a role in sustaining or exacerbating the degenerative process, potentially through mechanisms related to cellular stress responses or inflammatory pathways. Understanding the exact function of *PRLΔE1* in these contexts could provide valuable insights into its potential as a therapeutic target for IRDs.

While the precise modifications and transcription factors involved in *PRLΔE1* expression are currently under investigation, alternative Transcription Start Sites (aTSS) have been identified in 52% of human protein-coding genes [48]. Along with alternative splicing, aTSS significantly increase transcriptomic diversity, allowing for dynamic fine-tuning of the cellular transcriptome in response to various physiological and pathological stimuli [49–55]. Epigenetic alterations such as DNA methylation and histone acetylation/deacetylation are common to many neurodegenerative diseases, including IRDs, and change the chromosomal accessibility to direct transcription from aTSS [56–58]. We speculate that these epigenetic changes in IRDs may also direct the choice of aTSS in the *PRL* gene, thus inducing and upregulating *PRLΔE1* as disease progresses.

While we had previously found that *PRLΔE1* expression was associated with disease, it remained to be determined if it played an active role in IRD or its expression was a consequence of the disease process. Our *PRLΔE1* knockdown studies support a role for this transcript in promoting disease in the retina. This is in contrast with the anti-apoptotic role identified for the full length PRL in retina [10, 13], and the neurogenic and neuroprotective roles in the CNS [59]. Given the limitations of our currently used shRNA_*PRLΔE1*_ with demonstrated off-target effects even in normal retinas, we could not address in the present study the long-term effects that silencing *PRLΔE1* expression may have on PR preservation. Efforts to develop a *PRLΔE1* targeting shRNA without off target /non-specific deleterious effects are ongoing.

It is currently unclear whether *PRLΔE1* exerts its effect as RNA or protein. In our earlier publication [23], we showed that very small amounts of PRL protein could be identified in extracts from *PDE6β*-RCD1 and *RPGR*-XLPRA2 retinas using mass spectrometry. However, the low protein abundance and resulting poor peptide coverage precluded identification of the complete PRLΔE1 protein sequence. *PRLΔE1* mRNA lacks a consensus Kozak sequence [60] at the first in-frame AUG codon, potentially explaining the extremely low levels of PRLΔE1 protein in extracts from mutant retinas. However, the protein structure prediction algorithm I-TASSER [61, 62] estimates that the short protein isoform can form a 4 helix bundle similar to the full length PRL, and we predict that, if expressed, the protein should be able to bind the PRL receptor, albeit with lower affinity [23].

As *PRLΔE1* protein was present at extremely low levels if present at all, it is also worth considering the potential role of *PRLΔE1* mRNA as a regulatory non-coding RNA. Long non-coding RNAs (lncRNA) are abundantly expressed in CNS and neuroretina, have important roles in normal physiology and homeostasis, and are implicated in pathophysiology of neurodegenerative disorders including Alzheimer’s and Parkinson’s diseases, as well as in retinal degenerations [63–67]. Transcripts longer than 500 nucleotides (earlier definition included transcripts >200 nt) [68] can be considered lncRNAs and at >850 nt, *PRLΔE1* transcripts satisfy this first criterion. The second criterion for a transcript to be designated ncRNA is the exclusion of the defining characteristics of protein coding loci. However, as shown recently for many other lncRNAs [68], the *PRLΔE1* transcript exhibits most attributes of mRNAs. As we cannot currently rule out the potential for this transcript to have both protein coding and regulatory functions [68, 69], our ongoing studies are aimed at defining the active *PRLΔE1* entity.

We acknowledge two limitations in this study: the small number of dogs in which ONL rescue was demonstrated by *PRLΔE1* silencing and the lack of elucidation of the biological pathways through which *PRLΔE1* may drive rod degeneration and loss. We intentionally limited the number of dogs recruited for this study after we observed off-target effects with the currently used shRNA. Further, *PRLΔE1* isoform is not expressed in the rodent retinas [23], making dogs affected with IRDs valuable animal models in which the function of *PRLΔE1* can be investigated. Future studies employing more specific knockdown of this isoform will aim to identify the pathways that are activated by *PRLΔE1* in mutant retinas, and explore the translational potential of modulating its expression.

In summary, examination of *PRLΔE1* mRNA expression in four different forms of canine IRD with different degeneration paradigms reveals its consistent expression in IRDs, regardless of the causative gene mutation. Moreover, a causative role for *PRLΔE1* mRNA in disease progression is supported by our findings that expression is suppressed when disease progression is halted by corrective gene-specific therapy and that *PRLΔE1* knockdown in at least two models of IRD has an initial protective effect on the PRs. Together, our findings suggest that *PRLΔE1* knockdown/silencing represents an exciting novel “gene agnostic” therapeutic strategy for IRD. Future studies will include assessing the long-term outcome of *PRLΔE1* knockdown on preservation of PRs and identification of the specific pathways through which *PRLΔE1* modulates PR survival.

## Materials and methods

### Animal use statement

The studies adhered to the ARVO statement for the Use of Animals in Ophthalmic and Vision Research and were approved by the Institutional Animal Care and Use Committee of the University of Pennsylvania. All dogs were bred and maintained under standard care conditions (diet, ambient illumination with cyclic 12 h ON-12 h OFF light) at the Retinal Disease Studies Facility (RDSF) of the University of Pennsylvania. In compliance with the 3 R guiding principle, most studies were carried out on archival tissue sections from dogs that were previously used in other studies. All dogs used are listed in Supplementary Table 1.

### In vitro *PRLΔE1* shRNA screening

Three shRNA constructs driven by the H1-promoter and targeting different regions of the *PRLΔE1* were screened in vitro in HEK293 cells overexpressing the *PRLΔE1*-mCherry reporter gene. Briefly, HEK293 cells were seeded in 12 well plates and co-transfected the following day at 70–90% confluence with the *PRLΔE1*-mCherry plasmid and one of the three *PRLΔE1*-targetting shRNAs, or a non-specific shRNA. Transfections were performed in triplicates using Lipofectamine 2000. The cells were incubated for 24 h at 37 °C in a 5% CO_2_ with room air incubator. Following incubation, the cells were collected, total RNA extracted using the Qiagen RNeasy Plus Mini kit and reverse transcribed into cDNA using the High-Capacity cDNA Reverse Transcription kit (Applied Biosystems, ThermoFisher Scientific). qPCR analysis was performed using PowerUp SYBR Green Mastermix (Applied Biosystems, ThermoFisher Scientific) in either Applied Biosystems 7500 Real Time PCR System or Applied Biosystems ViiA 7 Real Time PCR System in a 96-well or 384-well format.

### Subretinal AAV-shRNA_*PRLΔE1*_ injections

The most efficient shRNA identified by in vitro screening was packaged in AAV2/5 (Ocular Gene Therapy Core, University of Florida, Gainesville, FL) and delivered subretinally in the superotemporal quadrant using a 25/38 G PolyTip cannula (MedOne^®^ Sarasota, FL, U.S.A.) attached to a 1 mL Luer-Lok™ syringe (Beckton, Dickinson and Company, NJ, U.S.A.) in dogs under general anesthesia. Injections were performed using a Stellaris PC injection system (Bausch and Lomb, Rochester, NY, USA) and visualized through a Zeiss operating microscope (Carl Zeiss Meditec, Inc., Dublin, CA, U.S.A.) with the image projected on a NGenuity^®^ 3D screen (Alcon, Geneva, Switzerland). Optimal dose of AAV2/5-shRNA_*PRLΔE1*_ was determined in a pilot study by subretinally injecting 150 µL of two different doses (5 × 10^11^ and 5 × 10^12^ vg/mL) of the vector in each eye of one *RPGR*-XLPRA2 dog (age ~40 weeks) when ample *PRLΔE1* expression in the PRs is observed. Subsequently, AAV2/5-shRNA_*PRLΔE1*_ was delivered at a dose of 5 × 10^12^ vg/mL in one *PDE6β*-RCD1 (age: 5 weeks) and two *RPGR*-XLPRA2 (age: 7 weeks) dogs (subretinal, volume: 70 µLs), and in one normal adult (age: 4.5 years) dog (subretinal, volume: 150 µLs).

### In vivo retinal imaging

Confocal scanning laser ophthalmoscopy and spectral domain optical coherence tomography (cSLO/OCT) was performed for in vivo retinal assessment (HRA + OCT Spectralis^®^, Heidelberg Engineering Inc, MA, USA). Autofluorescence cSLO images were taken using a 130° field-of-view lens, and 55° infra-red and blue-light. Sequential OCT scans of the bleb and bleb-adjacent areas were obtained to check for retinal changes over time. Further post-acquisition processing of cSLO/OCT data was performed with custom programs (MATLAB; MathWorks, Natick, MA, USA) as previously described [42, 70]. For retina-wide topographic analysis, integrated backscatter intensity of each raster scan was used to locate its precise location and orientation relative to retinal features visible on cSLO images. Individual longitudinal reflectivity profiles (LRPs) forming all registered raster scans were allotted to regularly spaced bins (1° × 1°) in a rectangular coordinate system centered at the optic nerve; LRPs in each bin were aligned and averaged. Intraretinal peaks and boundaries corresponding to the ONL were segmented using both intensity and slope information of backscatter signal along each LRP. For all topographic results, locations of blood vessels, optic nerve head, and bleb boundaries were overlaid for reference.

### Ocular tissue collection

Ocular tissues were collected after euthanasia with intravenous sodium pentobarbital injection. After enucleation, 3 mm diameter retinal punches were obtained within and outside the area injection bleb. The posterior eyecup was fixed at 4 °C in 4% paraformaldehyde (PFA) for 3 h, followed by 2% PFA for 24 h. Tissues were dissected, cryopreserved by sequential 24 h steps in 15% and 30% sucrose-PBS solutions, and then embedded in Optimal Cutting Temperature (OCT) medium.

### RNA in situ hybridization, immunohistochemistry, and TUNEL Assay

*PRLΔE1* mRNA was visualized by RNA-in situ hybridization (RNA-ISH) using the RNAscope assay 2.5 HD Assay-Red (Advanced Cell Diagnostics, ACD-Bio, BioTechne, Newark, CA, USA) as described previously [23]. Briefly, 10 µm thick sections were cut from PFA-fixed OCT-embedded canine retinas. Target retrieval was performed by heating the slides at 88 °C for 10 min in Target Retrieval buffer, followed by 15 min of protease treatment. *PRLΔE1* probe (RNAscope cat no. 535781) binding and visualization were performed as described in the kit protocol. Slides were then either counterstained with hematoxylin or used for immunohistochemistry/TUNEL assay. RNAscope 2.5 HD Duplex Assay was performed following kit recommendations for co-visualization of *PRLΔE1* and *NPHP5* (RNAscope probe cat no. 462841) RNAs. *PRLΔE1* was visualized using Fast Red dye and *NPHP5* was stained with HRP-Green.

When performing dual staining with RNA-ISH and immunohistochemistry (IHC), the retinal sections were treated with a blocking buffer for 1 h (5% BSA and 4.5% fish gelatin in PBS) immediately following RNA-ISH, and then incubated overnight with the primary antibodies (RHO, Millipore MAB5316, 1:200; ARR3, C.Craft, Univ. of Southern California, LUMIF; RPGR, Sigma HPA001593, 1:100) at 4 °C. Antigen antibody complexes were visualized with Alexa-stained secondary antibodies. Hoechst 33342 stain (Thermo Fisher Scientific) was used to label cell nuclei. TUNEL assay was performed to assess cell death in the retinal sections as previously reported [39]. Slides were mounted in Gelvatol mounting medium and examined with an epifluorescence microscope (Axioplan, Carl Zeiss Meditec). Images were captured with the Axiocam 305 camera and Zeiss Zen Microscopy software and processed using the Adobe Photoshop and Illustrator programs for display.

## Supplementary information

Supplementary Material

## Data Availability

All experimental data related to this study are included in the published article and supplementary materials. Any additional supporting data will be available from the corresponding authors upon request and shared on Dryad.
