# Supplementary material for: Retinal prolactin isoform PRLΔE1 sustains rod disease in inherited retinal degenerations
Source: Cell Death Dis. 2024 Sep 18;15(9):682. doi: 10.1038/s41419-024-07070-1 (PMC11410941; doi:10.1038/s41419-024-07070-1)
Supplement: Supplementary file 1 — Supplementary Material [file 41419_2024_7070_MOESM1_ESM.pdf]

Supplementary Table 1: List of dogs examined or used in this study

Dog ID	Genotype	Age	Sex	Experiment	Original Study/Treatment	Reference
H483	*RPGR*-XLPRA1 affected	77 wks	M	ISH for *PRLΔE1*	Untreated control eye for AAV-*RPGR* gene therapy	Beltran et al, 2012
H427	*RPGR*-XLPRA1 affected	6 wks	M	ISH for *PRLΔE1*	None	NA
H428	*RPGR*-XLPRA1 affected	9 wks	F	ISH for *PRLΔE1*	None	NA
H458	*RPGR*-XLPRA1 affected	16 wks	M	ISH for *PRLΔE1*	None	NA
H489	*RPGR*-XLPRA1 affected	26 wks	F	ISH for *PRLΔE1*	None	NA
H481	*RPGR*-XLPRA1 affected	37 wks	F	ISH for *PRLΔE1*	None	NA
AS2-407	*NPHP5*-LCA affected	33 wks	F	ISH for *PRLΔE1*	Untreated control eye for AAV-*NPHP5* gene therapy	Aguirre et al, 2021
AS278	*NPHP5*-LCA affected	6 wks	M	ISH for *PRLΔE1*	Untreated control eye for AAV-*NPHP5* gene therapy	Aguirre et al, 2021
AS2-414	*NPHP5*-LCA affected	9 wks	M	ISH for *PRLΔE1*	None	NA
AS280	*NPHP5*-LCA affected	14 wks	M	ISH for *PRLΔE1*	Disease characterization in *NPHP5* affected	Downs et al, 2016
AS281	*NPHP5*-LCA affected	32 wks	M	ISH for *PRLΔE1*	Disease characterization in *NPHP5* affected	Downs et al, 2016
AS282	*NPHP5*-LCA affected	42 wks	M	ISH for *PRLΔE1*	Disease characterization in *NPHP5* affected	Downs et al, 2016
GS214	*CNGB3*-ACHM3 affected	>5 yrs	F	ISH for *PRLΔE1*	None	NA
EM248	$RHO^{T4R/+}$ ADRP	38 wks	M	ISH for *PRLΔE1*	Exposure to 1mW/cm^2 light for 60s, PE interval 24 hours	Sudharsan et al, 2017
EM179	$RHO^{T4R/T4R}$ ADRP	73 wks	F	ISH for *PRLΔE1*	Exposure to 1mW/cm^2 light for 60s, PE interval 2 weeks	Sudharsan et al, 2017
EM181	$RHO^{T4R/T4R}$ ADRP	73 wks	F	ISH for *PRLΔE1*	Exposure to 0.5mW/cm^2 light for 60s, PE interval 24 hours	Sudharsan et al, 2017

EM186	$RHO^{T4R/T4R}$ ADRP	67 wks	F	ISH for *PRLΔE1*	Exposure to 0.5mW/cm^2 light for 60s, PE interval 2 weeks	Sudharsan et al, 2017
EM187	$RHO^{T4R/T4R}$ ADRP	67 wks	F	ISH for *PRLΔE1*	Exposure to 0.3mW/cm^2 light for 60s, PE interval 24 hours	Sudharsan et al, 2017
EM198	$RHO^{T4R/T4R}$ ADRP	55 wks	F	ISH for *PRLΔE1*	Exposure to 0.3mW/cm^2 light for 60s, PE interval 2 weeks	Sudharsan et al, 2017
1845	*PDE6β*-RCD1 affected	14 wks	M	ISH for *PRLΔE1*	None	NA
H414	*RPGR*-XLPRA1 carrier	24 wks	F	ISH for *PRLΔE1*	Disease characterization in *RPGR* carrier	Beltran et al, 2009
Z184	*RPGR*-XLPRA2 carrier	26.1 wks	F	ISH for *PRLΔE1*	Disease characterization in *RPGR* carrier	Beltran et al, 2009
H484	*RPGR*-XLPRA1 affected	77 wks	F	ISH for *PRLΔE1* after gene therapy	Eye treated with AAV-*RPGR* (age 28 wks), 49 wks PI	Beltran et al, 2012
Z412	*RPGR*-XLPRA2 affected	38 wks	M	ISH for *PRLΔE1* after gene therapy	Eye treated with AAV-*RPGR* (age 5 wks), 33 wks PI	Beltran et al, 2012
AS2-407	*NPHP5*-LCA affected	33 wks	F	ISH for *PRLΔE1* after gene therapy	Eye treated with AAV-*NPHP5* (age 6 wks), 27 wks PI	Aguirre et al, 2021
Z714	*RPGR*-XLPRA2 affected	46.1 wks	F	AAV-shRNA$_{PRLΔE1}$ dosage optimization	-	Current study
2369	*PDE6β*-RCD1 affected	14 wks	F	AAV-shRNA$_{PRLΔE1}$ treatment, 9 wks PI	-	Current study
Z731	*RPGR*-XLPRA2 affected	12.9 wks	M	AAV-shRNA$_{PRLΔE1}$ treatment, 5.7 wks PI	-	Current study
Z730	*RPGR*-XLPRA2 affected	19 wks	M	AAV-shRNA$_{PRLΔE1}$ treatment, 11.9 wks PI	-	Current study
N329	Normal	4y 8m	M	shRNA$_{PRLΔE1}$ treatment, 8.1 wks PI	-	Current study

PE: Exposure interval; PI: Post AAV injection

Supplementary Figure 1

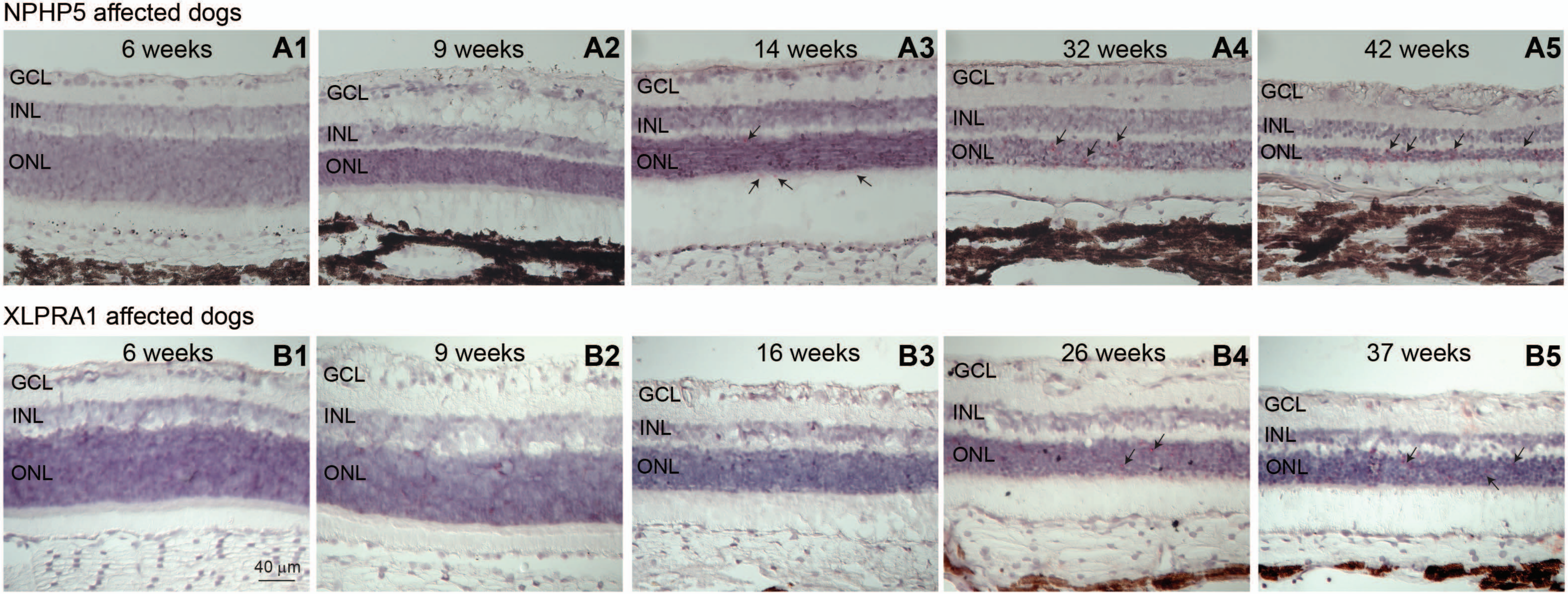

Supplementary Figure 1: Induction of PRLΔE1 expression in NPHP5-LCA and RPGR-XLPRA1 retinas with disease progression. **(A1-B5)** Photomicrographs of H&E stained retinal cryosection labeled with RNA-ISH to visualize PRLΔE1 expression in the NPHP5-LCA **(A1-A5)** and RPGR-XLPRA1 **(B1-B5)** dogs at various ages. One dog was used for each genotype and age.
ONL: Outer nuclear layer; INL: Inner nuclear layer; GCL: Ganglion cell layer.

Supplementary Figure 2

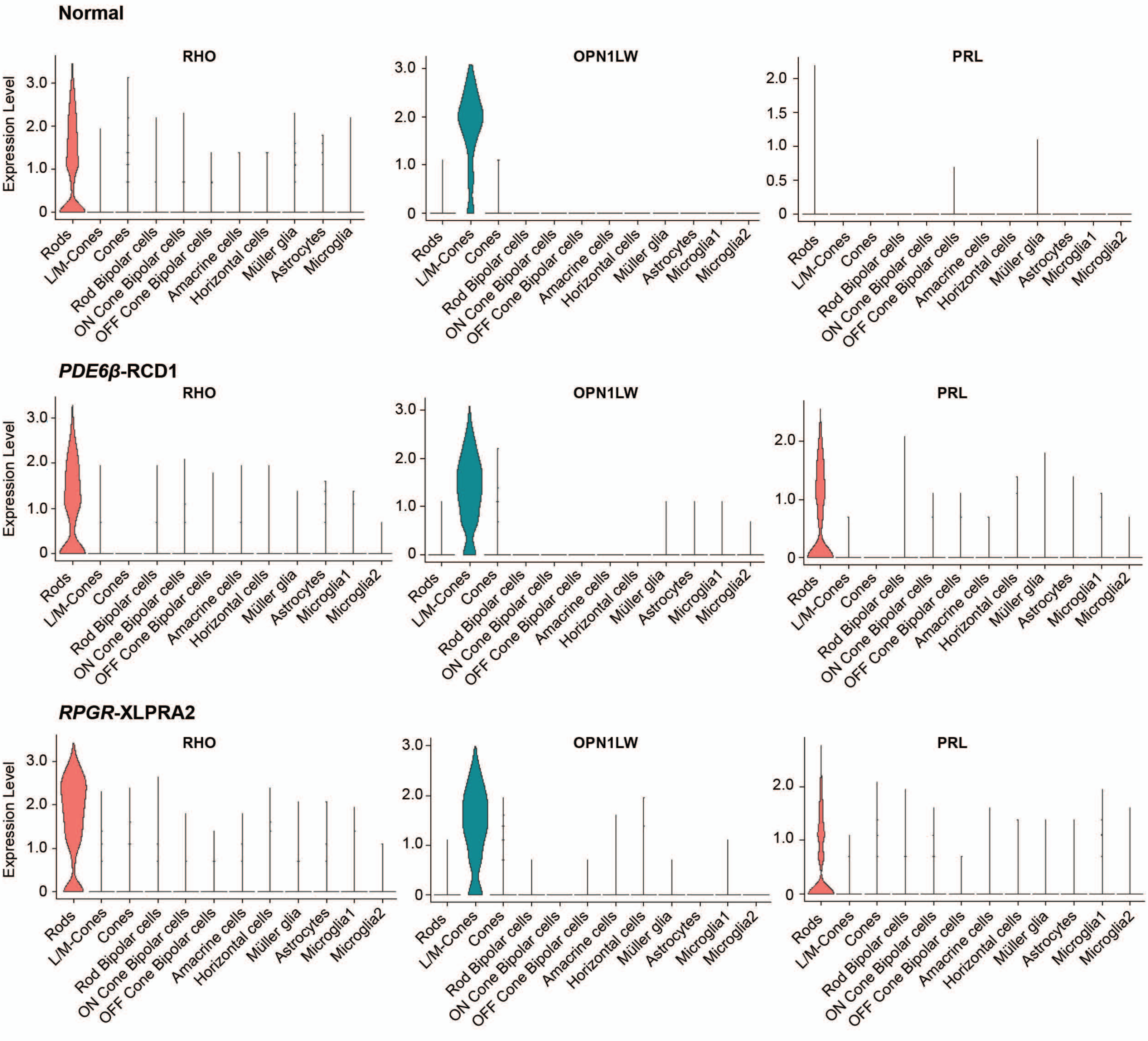

Supplementary Figure 2: Rods but not cone PRs express PRLΔE1. Single cell RNAseq data showing RHO, OPN1LW and PRLΔE1 expression in PDE6β-RCD1 and RPGR-XLPRA2 retinas. Representative data from two dogs for each genotype.

Supplementary Figure 3

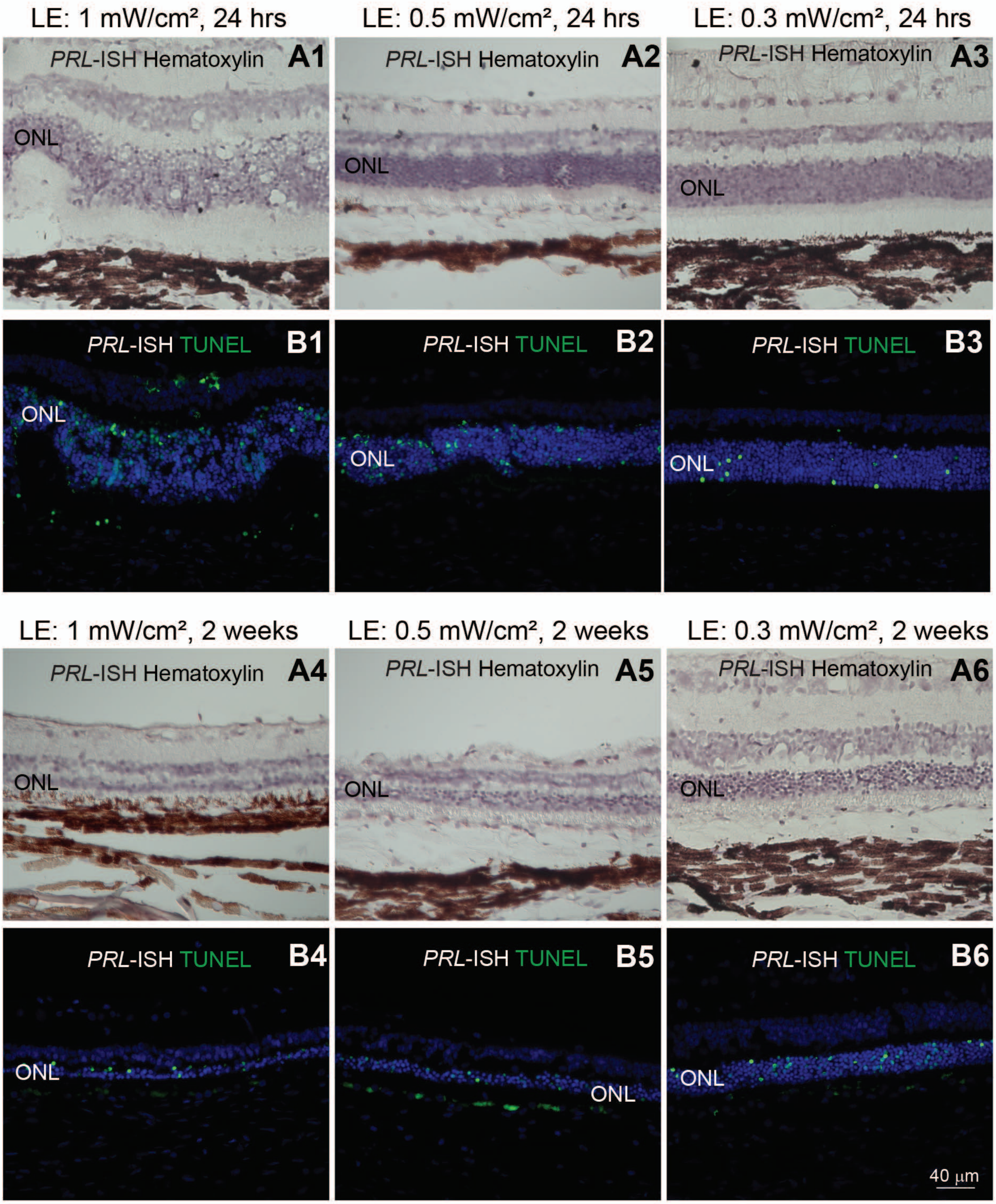

Supplementary Figure 3: Expression of PRLΔE1 isoform is not observed in the rapidly degenerating RHO-T4R retinas post light exposure (LE). (A1-A6) Photomicrographs of H&E stained retinal cryosections labeled with RNA-ISH to visualize PRLΔE1 expression at 24 hours **(A1-A3)** and 2 weeks **(A4-A6)** post light exposure (LE) to 3 different intensities. **(B1-B6)** Photomicrographs of retinal cryosections co-labeled with TUNEL (green) and RNA-ISH for PRLΔE1 at 24 hours **(B1-B3)** and 2 weeks **(B4-B6)** post light exposure (LE) to 3 different intensities. Representative images from one dog assessed per time point and LE condition.

Supplementary Figure 4

A

```
ATGTTCAACGAATTTGATAAAAGGTATGCCCAGGGCCGGGGGTTCATTACCAAGGCCATCAACA
GCTGTCACACCTCCTCCCTCTCTACCCCTGAAGACAAGGAGCAAGCCCAACAGATCCACCATGA
AGACCTTCTGAATCTGATACTGAGGGTGCTGCGCTCCTGGAATGACCCCCTGTATCATCTAGTC
ACAGAAGTGCGGGGGATGCAAGAAGCCCCAGATGCAATTCTATCCAGAGCCATAGAGATTGAAG
AACAAAACAGAAGACTTCTAGAGGGTATGGAGAAGATAGTTGGCCAGGTTCATCCTGGAATCAG
AGAAAATGAGGTCTACTCTGTCTGGTCAGGACTTCCATCCCTGCAGATGGCGGATGAAGACACT
CGCCTTTTTGCTTTTTATAACCTGCTCCACTGCCTACGCAGGGATTCACATAAGATTGACAATT
ATCTCAAGCTCCTGAAGTGCCGAATCGTCTACGACAGCAACTGCTAA
```

B

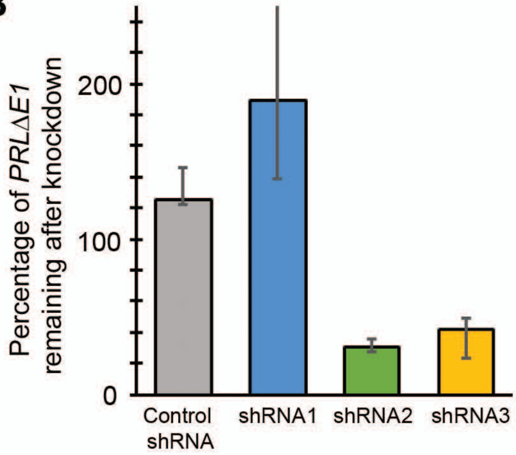

Supplementary Figure 4: Identification of optimal shRNA for PRLΔE1 knockdown. (A) PRLΔE1 sequence with the three shRNA target sequences highlighted. **(B)** qPCR analysis data comparing the percentage of transcript remaining in HEK293 cells co-transfected with pCMV-Tag5a PRLΔE1 and one of the three PRLΔE1-targeting shRNAs or a non-specific control shRNA (n = 3). shRNA2 was selected for in vivo silencing studies.
